# The Synergistic Effect of Heat Therapy and Electroacupuncture Treatment in Inflammatory Pain Mouse Models

**DOI:** 10.3390/brainsci15080822

**Published:** 2025-07-31

**Authors:** Boon Khai Teoh, Sharmely Sharon Ballon Romero, Tran Van Bao Quach, Hsin-Yi Chung, Yi-Hung Chen

**Affiliations:** 1 Graduate Institute of Acupuncture Science, China Medical University, No. 91, Hsueh-Shih Road, Taichung 404328, Taiwan; pulau1959@gmail.com (B.K.T.); sharmely@hotmail.com (S.S.B.R.); qvbt1205@gmail.com (T.V.B.Q.); mow3607@gmail.com (H.-Y.C.); 2 International Master Program in Integrative Health, China Medical University, Taichung City 404328, Taiwan; 3 Chinese Medicine Research Center, China Medical University, Taichung City 404328, Taiwan

**Keywords:** heat therapy, modern moxibustion, electroacupuncture, adenosine pathway, opioid receptors, astrocytes

## Abstract

**Background:** Heat therapy (HT) and electroacupuncture (EA) are widely utilized pain relief methods, but the analgesic mechanisms of their combined application remain unclear. **Methods:** In acetic acid (AA)-induced writhing test and complete Freund’s adjuvant (CFA)-induced inflammatory pain tests, mice received one of three treatments: EA at bilateral ST36, HT via a 45 °C heating pad, or the combination (EA + HT). To probe underlying pathways, separate groups were pretreated with caffeine, DPCPX (a selective adenosine A_1_ receptor antagonist), or naloxone (an opioid receptor antagonist). Spinal expression of glial fibrillary acidic protein (GFAP) and phosphorylated p38 (p-p38) was examined by Western blot and immunofluorescence. **Results:** Both EA and HT individually reduced AA-induced writhing, with the combination (EA + HT) exhibiting the greatest analgesic effect. EA’s analgesic effect was reversed by caffeine and DPCPX and partially by naloxone, while HT’s effect was reversed by caffeine and DPCPX but was unaffected by naloxone. AA injection elevated spinal p-p38 and GFAP expression, which were attenuated by either EA or HT, with the most substantial suppression observed in the EA + HT group. In the CFA model, both treatments alleviated mechanical allodynia, while the combined treatment resulted in significantly greater analgesia compared to either treatment alone. **Conclusions:** EA combined with HT synergistically enhances analgesia in both AA and CFA pain models, accompanied by reduced spinal inflammation and astrocyte activation. EA’s analgesic effects appear to involve adenosine A_1_ receptor pathways and, to a lesser extent, opioid receptor mechanisms, whereas HT’s effects involve adenosine A_1_ receptor pathways.

## 1. Introduction

Pain is a multifaceted phenomenon and one of the most prevalent medical complaints, significantly impacting individuals’ quality of life [1]. Pain is defined by the International Association for the Study of Pain (IASP) as “An unpleasant sensory and emotional experience associated with, or resembling that associated with actual or potential tissue damage” [1]. Pain can be classified temporally as acute or chronic, and mechanistically as nociceptive—resulting from activation of peripheral nociceptors, neuropathic—arising from lesions or diseases of the somatosensory system, and nociplastic—defined as pain arising from altered nociception not fully explained by the other two mechanisms [2]. Acute nociceptive pain triggered by noxious stimuli such as heat, cold, mechanical force, or chemical agents may progress to inflammatory and chronic pain in 10.1–55.2% of cases if not treated early [3], since acute pain becomes inflammatory pain when persistent nociceptor activation releases pro-inflammatory markers that sensitize local and central pathways, driving glial activation and central sensitization [4,5]. Neuroinflammation—marked by activation of glial cells, especially astrocytes, and microglia—contributes to central sensitization and can prolong nociceptive pain via dysregulated glial functions [6,7]. Activated astrocytes upregulate glial fibrillary acidic protein (GFAP), the hallmark intermediate filament of reactive astrocytes, and secrete pro-inflammatory cytokines that further enhance nociceptive transmission [6]. Despite advances in understanding pain mechanisms, effective and side-effect-free therapies for managing pain, especially in the acute stage to prevent transformation to chronic pain, are still needed.

Acupuncture, a traditional Chinese medicine (TCM)-based technique involving the insertion of needles at specific acupoints, has been widely used for pain management [8]. It involves the stimulation of the acupoint at different layers and frequencies, which triggers the release of endogenous opioids and other neurotransmitters [9].

Heat therapy (HT) is the application of an external heat source to a specific body region to elevate local tissue temperature and elicit therapeutic effects, using modalities such as hot packs, hydrothermal baths, therapeutic ultrasound, infrared lamps, and saunas with adjustable temperature settings [10,11,12,13,14,15]. By tuning the temperature, different thermoTRP receptors are selectively engaged—TRPV1 opens at approximately ≥42 °C and TRPV2 at ≥52 °C—while higher, noxious heat can activate polymodal nociceptors. Activation of these channels triggers a cascade of downstream mechanisms: vasodilation with increased shear stress [16,17], reduced muscle spindle sensitivity and trigger-point activity [18,19,20], modulation of metabolic regulators like irisin and adiponectin [21], induction of heat shock proteins (notably HSP70) [22], therefore inducing anti-inflammatory and analgesic effects.

In terms of heat therapy, moxibustion, one major treatment method in TCM, can also be classified into this group of treatments. A moxa stick made of mugwort (*Artemisia argyi*) utilizes heat to stimulate acupoints and achieve therapeutic effects [23,24,25]. Many reports have provided great evidence that acupuncture and moxibustion together have the greatest pain relief effect compared to each alone [26,27]. However, there is a lack of information about the combination of acupuncture and other heat therapy (acupuncture combined with a hot pad, infrared light, etc.), except warm acupuncture, which directly increases the temperature of the acupuncture needle.

The adenosine A_1_ receptor (A_1_R) is one of four G protein-coupled adenosine receptor subtypes that bind the endogenous ligand adenosine and couple to Gᵢ/o proteins, resulting in inhibition of adenylate cyclase, reduced cAMP levels, blockade of Ca^2^⁺ channels, and activation of K⁺ currents [28]. It is broadly expressed in the central nervous system, heart, vasculature, and other tissues—where it mediates inhibitory effects such as analgesia, sedation, and negative chronotropic actions on the heart [28]. Additionally, A_1_R activation has been linked to reduced glial cell activation and neuroinflammation, providing a potential dual mechanism for pain relief [29]. Recent studies suggest that adenosine, a neuromodulator released during acupuncture, activates A_1_Rs to hyperpolarize nociceptive neurons and suppress C-fiber signaling, thereby diminishing pain [29,30,31]. Moxibustion also increases the level of ATP to induce analgesic effects [32]. However, the correlation between heat therapy and the adenosine pathway remains unclear.

The acetic acid (AA)-induced writhing test (AA model) in mice is used to study the nociceptors lining the peritoneum and measure spontaneous pain. Acetic acid triggers the release of inflammatory mediators (e.g., prostaglandin E2, bradykinin, substance P), which activate peripheral nerve endings to induce a pain response, represented by the number of writhing events (stretching, retracting, or pressing the belly against the floor), modeling the characteristics of human pain [33].

Accordingly, this study aimed to investigate the analgesic mechanisms of heat therapy, applied via a heating pad, and to determine whether it exerts overlapping or synergistic effects when combined with electroacupuncture (EA) in an acute pain model induced by AA injection. Furthermore, the involvement of adenosine and opioid receptor pathways was examined to clarify the underlying mechanisms.

We additionally applied a complete Freund’s adjuvant (CFA)-induced inflammatory pain model (CFA model), in which mechanical allodynia was assessed using the von Frey test [34,35]. This second model served to confirm whether the analgesic effects of EA, HT, and their combination are also observed in a somatic inflammatory context involving peripheral hypersensitivity.

## 2. Materials and Methods

### 2.1. Animals

ICR male mice (25–30 g; 4-5 weeks old, BioLasco Taiwan Co., Ltd., Taipei, Taiwan) were used for the AA model, while C57BL/6 male mice (20-25g, 5-6 weeks old, BioLasco Taiwan Co., Ltd., Taipei, Taiwan) were used for the CFA model. The mice were housed under standard conditions at a 12:12 light/dark cycle with food and water available ad libitum. Mice were randomly divided into groups (*n* = 9 in each group). The experimental procedures and housing conditions were according to the guideline issued by the China Medical University Institutional Animal Care and Use Committee, and issued by the Affidavit of Approval of Animal Use Protocol, China Medical University. The animal use approval numbers for the AA model were CMUIACUC-2022-364, CMUIACUC-2022-364-1, CMUIACUC-2022-364-2, and CMUIACUC-2022-364-3; for the CFA model, the approval number was CMUIACUC-2025-264.

### 2.2. Writhing Test with Acetic Acid

**Figure 1:**
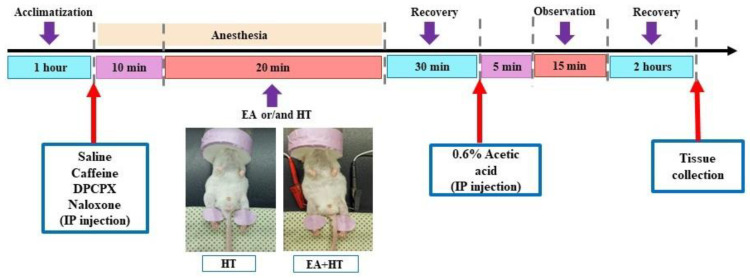
Experimental procedure chart.

In this study, the writhing test was performed as previously described methodology [36,37] and the procedure was illustrated in Figure 1. Animals were injected intraperitoneally (i.p.) with a 0.6% (*v*/*v*) solution of acetic acid (Aldrich Sigma, Saint Louis, MO, USA) in normal saline (10 mL/kg). Following the acetic acid injection, animals exhibited a stereotypic response pattern in the form of a stretching movement or “writhing”, which is interpreted as a pain-like response [37]. Five minutes after the acetic acid injection, the writhing response was recorded and counted for over 15 min.

### 2.3. CFA Induces Inflammatory Pain

Mice were anesthetized with 2% isoflurane and received intraplantar injections of complete Freund’s adjuvant (30 μL, 1 mg/mL heat-killed Mycobacterium tuberculosis from Sigma Chemical Company, St. Louis, MO, USA) into the left hind paw to induce localized inflammation. Animals were returned to their home cages and allowed to recover before behavioral testing [38].

### 2.4. Von Frey Test

Paw withdrawal thresholds (PWTs) were assessed using an electronic von Frey apparatus (EVF-3; Bioseb, Vitrolles, France) one day before CFA injection (baseline), immediately post-injection (CFA), and on Days 1 and 2 after treatment, following a previous study [38]. Mice were placed on an elevated mesh grid under individual plastic enclosures for 60 min habituation before testing. The test was conducted under blinded conditions. The filament was applied vertically to the plantar surface of each hind paw with enough force to ensure contact. Each paw was stimulated five times, and the mechanical withdrawal responses were recorded automatically in grams. The values of withdrawal thresholds from 5 measurement times were averaged.

### 2.5. Electroacupuncture (EA) Stimulation Procedures

Experimental animals that underwent EA stimulation alone or in combination (HT or drugs) were initially placed in an induction chamber with the oxygen flow meter adjusted to approximately 2.0 L/min for about 15 min [39]. To minimize restraint-induced stress in mice, the electroacupuncture session involved a mask connected to a non-rebreathing circuit (Bain) with 1.5% isoflurane (Panion & BF Biotech Inc., Taoyuan, Taiwan). EA procedure was applied using a stimulator (Trio-300; Ito, Japan) at an intensity of 150 μs square pulses of 2 mA at 2 Hz for 20 min. The acupuncture needles used were 36-gauge (Shanghai Yanglong Medical Articles Co., Ltd., Shanghai, China) [40].

A single session of EA treatment of about 20 min was conducted for each mouse. Bilateral Zusanli (ST36) acupoints were selected for EA treatment. To locate the ST36 acupoint, we first ubicate the ST35 acupoint, which is located at the depression lateral to the patella ligament. The ST36 is situated at the proximal one-fifth point on the line from ST35 to the anterior side of the ankle crease. For this study, the anatomical location of the distal acupoints was determined according to the trans-positional acupoint system previously described [37,40,41]. Mice in the control group were placed under isoflurane anesthesia, 1.5% for 30 min—the same duration as the EA, HT, or combined treatments—without receiving any stimulation.

### 2.6. Heat Therapy (Electric Heating Pad)

Heat therapy treatment was administrated via an electric heating pad (LD Lead, Beijing, China). The heating stimulation parameters of the heating pad were at a constant temperature of 45 °C. The procedure also involved a mask connected to a non-rebreathing circuit with 1.5% isoflurane and lasted for over 20 min. The electric heating pad was used to warm the unshaven mice’s hind legs during the different experimental procedures (Figure 1).

### 2.7. Drug Selection and Administration

The following drugs were administrated: Naloxone (at a dose of 10 mg/kg), Caffeine (at a dose of 10 mg/kg), or 1,3-dipropyl-8-cyclopentyl xanthine (DPCPX, at a dose of 30 mg/kg), based on its association with EA treatment and its different analgesic pathways [30,37]. Naloxone, caffeine, and DPCPX were purchased from Sigma Chemical Company (St. Louis, MO, USA). Each drug was diluted in saline to achieve the target dose per body weight (mg/kg) within a consistent injection volume of 10 mL/kg. For example, a 1 mg/kg dose was prepared as a 1 mg/mL solution, ensuring each mouse received an accurate dose volume.

### 2.8. Western Blot Analysis

Mice were deeply anesthetized and euthanized by decapitation. Spinal cord samples corresponding to the L1 and L2 segments were collected and stored at −80 °C for further processing.

The samples were homogenized in RIPA buffer containing proteinase inhibitors, and protein levels were evaluated using a Pierce BCA protein assay kit. Samples of approximately 30 μg of protein were prepared and loaded onto precast 12% SDS-PAGE gels. The PVDF membranes were blocked with 5% BSA in Tris-buffered PBS for 1 h at room temperature and incubated overnight at 4 °C with the following primary antibodies: mouse anti-GFAP (1:2000; #3670; CST), or rabbit anti-phospho-p38 (1:1000; #9211; CST). Signals were normalized to those of β-actin (1:10,000; GTX629630) or anti-p38 (1:10,000; #9212; CST) antibodies as an internal control, respectively. Chemiluminescent signals were digitally scanned using the FUSION FX software, version 16.07 (Vilber, Marne-la-Vallée, France).

### 2.9. Immunofluorescent (IF)

The L1-L2 section of the spinal cord was processed into 30 µm-transverse slices as previously reported [42]. The sections were suspended and blocked for 60 min with 10% donkey serum in PBS containing 0.4% Triton X-100 before being incubated overnight at 4 °C with a primary antibody mouse anti-GFAP (1:500; Cell Signaling Technology, Danvers, MA, USA, 3670). After three washes with PBS, the sections were incubated with a secondary antibody for 60 min at room temperature (1:250, Alexa Fluor donkey anti-mouse 488, Abcam, Cambridge, UK, ab150105; 1:250, washed, mounted (ProLong™ Diamond Antifade Mountant with DAPI, Thermo Fisher P36971, Waltham, MA, USA), and coverslipped. Fluorescent images were captured using a confocal microscope (Leica SP5 TCS; Heidelberg, Germany).

### 2.10. Statistical Analysis

All data are expressed as mean ± standard deviation (SD), and *p* values < 0.05 were considered statistically significant. Between-group comparisons, at each time-point, were performed using one-way ANOVA, followed by Holm–Šídák post hoc test. Data were analyzed using GraphPad Prism 9 (GraphPad Software, version 9.0.0.121, Inc., San Diego, CA, USA).

## 3. Results

### 3.1. Effects of Heat Therapy and Electroacupuncture, Alone and in Combination, in the AA-Induced Writhing Test

To test for synergy, mice received EA alone, HT alone, or EA + HT, with all treatments completed 30 min before intraperitoneal injection of 0.6% acetic acid (AA). Five minutes after AA administration, writhing behaviors were observed for 15 min.

**Figure 2:**
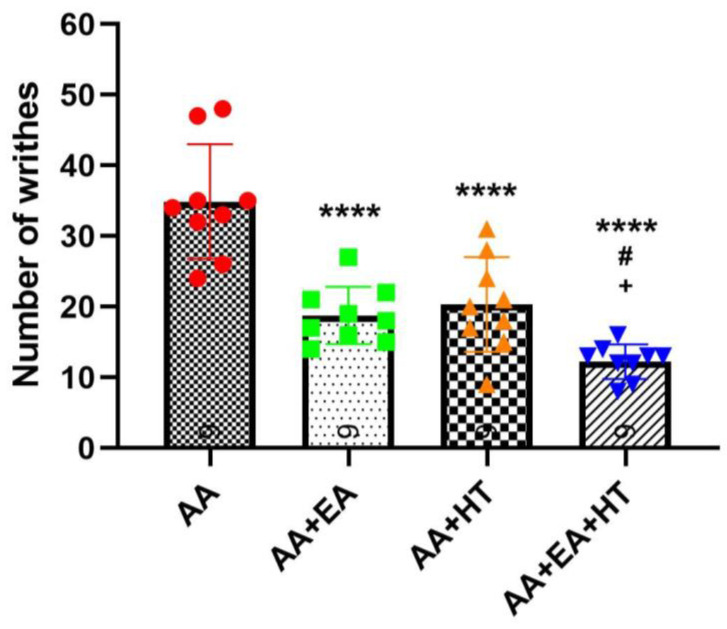
Evaluation of the analgesic effects of HT and EA, alone and in combination, therapy in AA-induced writhing test. Comparison of the effects of HT and EA, individually and combined, on behavioral test were performed. Data are presented as mean ± SD (*n* = 9 in each group). Between-group comparisons were performed by one-way ANOVA, followed by the Holm–Šídák post hoc test (**** *p* < 0.0001 vs. AA; ^#^ *p* < 0.05 vs. AA + EA, ^+^ *p* < 0.05 vs. AA + HT).

As expected, AA administration significantly induced writhing responses, with the AA group exhibiting 34.89 ± 8.13 writhes on average (Figure 2). Both EA and HT treatments individually led to substantial reductions in pain responses: the AA + EA group showed a significant decrease to 18.78 ± 4.06 writhes (*p* < 0.0001 vs. AA), while the AA + HT group exhibited an average of 20.33 ± 6.71 writhes (*p* < 0.0001 vs. AA). These results demonstrate that EA and HT independently provide considerable analgesic effects.

Importantly, the combination of EA and HT (AA + EA + HT group) further enhanced analgesia, reducing the writhing responses to 12.22 ± 2.44 on average. This combined effect was statistically significant when compared to the AA group (*p* < 0.0001) and superior to either EA or HT treatment alone (*p* < 0.05 vs. AA + EA and AA + HT). These data suggest a synergistic interaction between EA and HT in providing pain relief.

### 3.2. Behavioral Evaluation of the Effects of EA Alone or in Combination with Caffeine, DPCPX, and Naloxone in the AA-Induced Writhing Test

Previous studies have reported that acupuncture analgesia might involve interactions between adenosine and opioid receptor pathways [9,24,25]. To determine the involvement of adenosine and opioid receptors in EA-induced analgesia, we examined the effects of caffeine (CA; a non-selective adenosine receptor antagonist), DPCPX (DP; a selective adenosine A1 receptor antagonist), and naloxone (NA; an opioid receptor antagonist) in combination with EA using the AA-induced abdominal constriction mouse model. Five experimental groups were evaluated: AA, AA + EA, AA + CA + EA, AA + DP + EA, and AA + NA + EA. The experimental timeline followed the previously described protocol (Figure 1).

**Figure 3:**
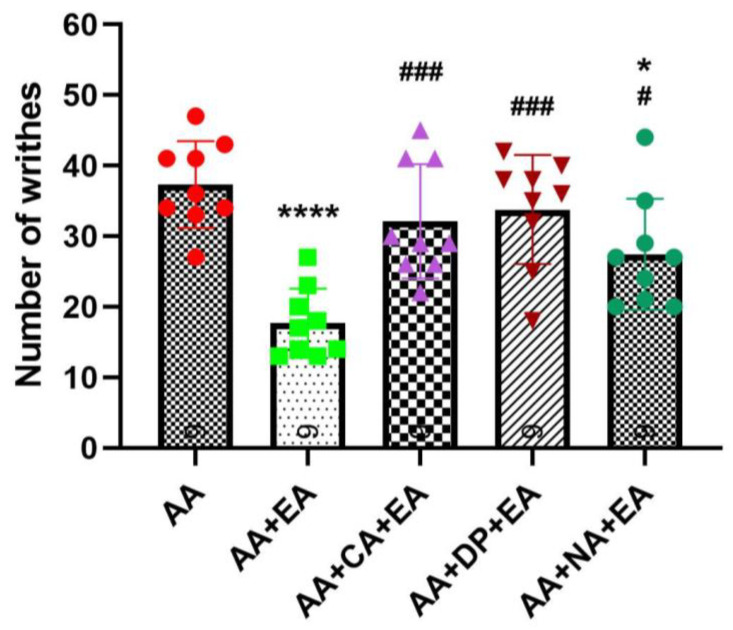
Comparison of the effects of EA, alone and in combination with caffeine (CA), DPCPX (DP), and naloxone (NA) in the AA-induced writhing test. Data are presented as the mean ± SD (*n* = 9 in each group). Between-group comparisons were performed by one-way ANOVA, followed by the Holm–Šídák post hoc test (* *p* < 0.05, **** *p* < 0.0001 vs. AA; ^#^ *p* < 0.05, ^###^ *p* < 0.001 vs. AA + EA).

Consistent with earlier findings (Figure 2), the injection of AA robustly induced writhing responses, with the AA group averaging 37.33 ± 6.14 writhes (Figure 3). EA treatment alone significantly reduced the number of writhes to 17.67 ± 4.90 writhes (*p* < 0.0001 vs. AA). Co-administration of EA with the opioid antagonist naloxone also produced a significant reduction in writhing responses to 27.44 ± 7.89 writhes (*p* < 0.05 vs. AA). However, this reduction was significantly less pronounced than that observed with EA treatment alone (*p* < 0.05 vs. AA + EA). These data suggest partial involvement of opioid receptors in EA-induced analgesia, although EA retains some analgesic efficacy despite opioid receptor blockade.

Notably, co-administration of EA with adenosine receptor antagonists, caffeine or DPCPX, markedly attenuated the analgesic effects of EA. Both the AA + CA + EA and AA + DP + EA groups showed significantly higher writhing responses (approximately 32.11 ± 8.10 and 33.78 ± 7.73 writhes, respectively) compared with the EA group (both *p* < 0.001 vs. AA + EA), and did not differ from the AA group. These results suggest an essential role of adenosine A1 receptor pathways in mediating the analgesic mechanisms of EA.

### 3.3. Effects of Heat Therapy, Alone or in Combination with Caffeine, DPCPX, and Naloxone in the AA-Induced Writhing Test

Similarly, we continued to evaluate the involvement of adenosine and opioid receptors in heat therapy (HT)-induced analgesia. The experimental groups included AA, AA + HT, AA + caffeine + HT (AA + CA + HT), AA + DPCPX + HT (AA + DP + HT), and AA + naloxone + HT (AA + NA + HT). The treatment schedule followed the same protocol described previously (Figure 1).

**Figure 4:**
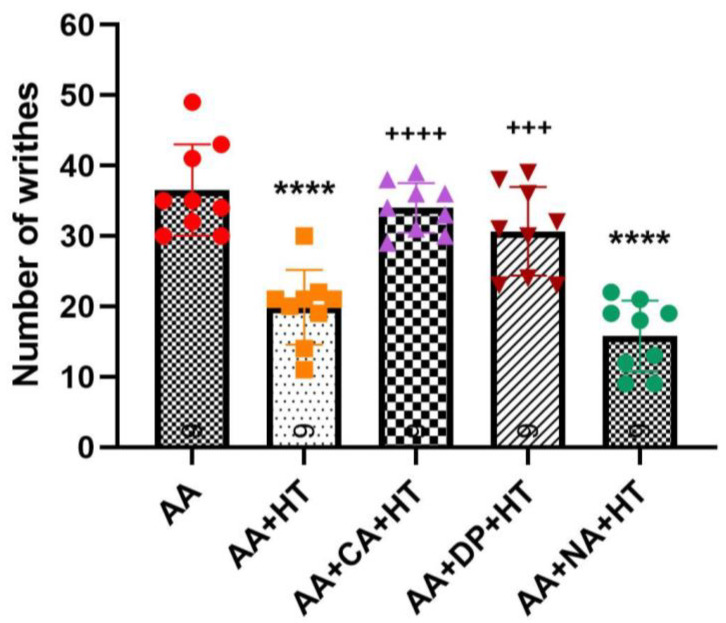
Combination of heat therapy, alone or in combination with caffeine (CA), DPCPX (DP), and naloxone (NA) in the AA-induced writhing test. Data are presented as the mean ± SD (*n* = 9 in each group). Between-group comparisons were performed by one-way ANOVA, followed by the Holm–Šídák post hoc test (**** *p* < 0.0001 vs. AA; ^+++^ *p* < 0.001, ^++++^ *p* < 0.0001 vs. AA + HT).

As shown in Figure 4, injection of 0.6% AA significantly induced writhing responses in the AA group, with an average of approximately 36.56 ± 6.46 writhes. Treatment with HT alone (AA + HT) significantly reduced the writhing responses to an average of 19.89 ± 5.30 writhes (*p* < 0.0001 vs. AA-only). Similarly, the AA + NA + HT group maintained significant analgesic effects, averaging 15.78 ± 5.07 writhes (*p* < 0.0001 vs. AA-only), and showed no significant difference compared to the AA + HT group. These results suggest that the analgesic effect of HT occurs independently of the opioid receptor pathway.

In contrast, both the AA + CA + HT and AA + DP + HT groups exhibited a full reversal of HT’s analgesic effects with no difference from the AA group. The AA + CA + HT group showed an average of approximately 34 ± 3.54 writhes, significantly higher compared to the AA + HT group (*p* < 0.0001). Similarly, the AA + DP + HT group displayed approximately 30.67 ± 6.29 writhes, also significantly higher than the AA + HT group (*p* < 0.001). These findings indicate that antagonism of adenosine A1 receptors by caffeine or DPCPX reversed HT’s analgesic mechanisms, suggesting a critical role for the adenosine A1 receptor pathway in mediating HT-induced analgesia.

### 3.4. p38 MAPK Activation Is Increased in the AA Group and Suppressed by EA and HT

**Figure 5:**
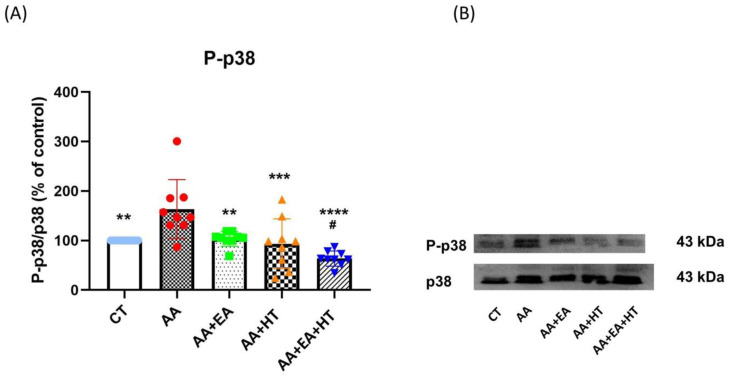
P-p38 protein expression in the AA-induced writhing test. (**A**) Quantitative analysis of phosphorylated p38 (p-p38) protein levels by Western blot, normalized to total p38 as a loading control. (**B**) Representative Western blot bands of p-p38 and total p38. Data are presented as the mean ± SD (*n* = 9 in each group). Between-group comparisons were performed by one-way ANOVA, followed by the Holm–Šídák post hoc test (** *p* < 0.01, *** *p* < 0.001, **** *p* < 0.0001 vs. AA; ^#^ *p* < 0.05 vs. AA + EA).

Several lines of evidence demonstrate that the stress-activated protein kinase p38 plays a crucial role in generating pain sensitivity [43]. Phosphorylated p38 (p-p38), the active form of p38, is increased following pain generation, and this activation leads to the production and release of proinflammatory cytokines [44]. To further confirm the modulatory effects of EA and HT on pain-related inflammation, we examined phosphorylation levels of p-p38 MAPK in the L1–L2 spinal cord two hours post-acetic acid (AA) injection using Western blot analysis (Figure 5).

In the AA group, p-p38 levels significantly increased to 161.5 ± 24.56% of the Control group (*p* < 0.05), indicating a robust inflammatory response. EA treatment alone (AA + EA group) significantly reduced p-p38 expression to 97.51 ± 18.00% of control (*p* < 0.01 vs. AA group). HT treatment alone (AA + HT group) also decreased p-p38 expression to 115.8 ± 49.26% of control (*p* < 0.01 vs. AA group). Notably, the combined EA and HT treatment (AA + EA + HT group) resulted in the greatest suppression, reducing p-p38 levels to 67.87 ± 11.99% of the Control group (*p* < 0.0001 vs. AA and *p* < 0.05 vs. AA + EA groups), clearly demonstrating a potent synergistic anti-inflammatory effect.

These findings strongly suggest that EA and HT synergistically inhibit p38 MAPK phosphorylation in the L1–L2 spinal cord.

### 3.5. EA and HT Reduce GFAP Expression, Indicating Astrocyte Activation Suppression

Following injury or inflammation, spinal dorsal horn astrocytes become reactive, characterized by hypertrophy, elevated expression of glial fibrillary acidic protein (GFAP), and release of pro-inflammatory mediators [40].

**Figure 6:**
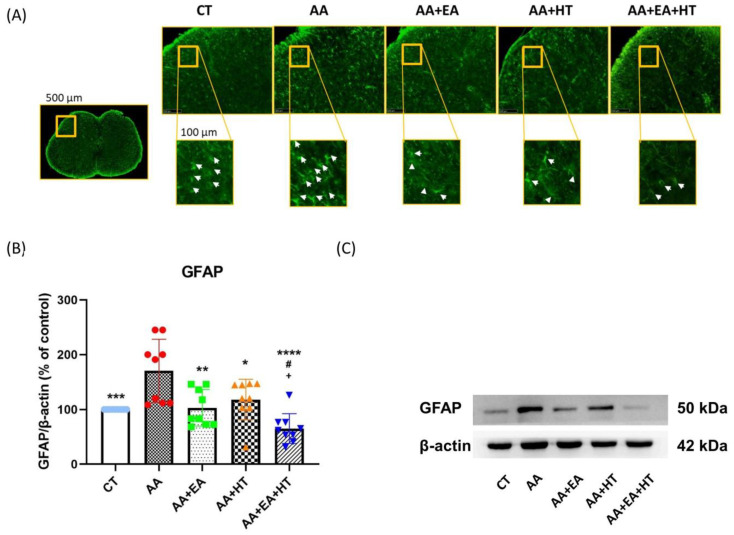
GFAP protein expression in the AA-induced writhing test. (**A**) Immunofluorescence staining of GFAP in the L1-L2 spinal cord region. Representative images show the expression of GFAP (green fluorescence) in the spinal cord sections of mice under different conditions. The yellow box in the overview image (left) indicates a field width of 500 µm, while all magnified inset boxes represent areas 100 µm in width. White arrows indicate activated GFAP-labeled cells, which are more prevalent in pain-induced conditions (AA group) and appear to be modulated in response to EA and HT treatments. (**B**,**C**) Western blot protein quantification and representative bands of GFAP, and β-actin as a loading control. Data are presented as the mean ± SD (*n* = 9 in each group). Between-group comparisons were performed by one-way ANOVA, followed by the Holm–Šídák post hoc test (* *p* < 0.05, ** *p* < 0.01, *** *p* < 0.001, **** *p* < 0.0001 vs. AA; ^#^ *p* < 0.05 vs. AA + EA, ^+^ *p* < 0.05 vs. AA + HT).

To further explore the effects of EA and HT on astrocyte activation, we assessed GFAP expression in the L1–L2 spinal cord using immunofluorescence staining (Figure 6A) and Western blotting (Figure 6B,C).

Immunofluorescence staining revealed a high density of GFAP-positive cells (white arrows) in the spinal dorsal horn of mice treated with acetic acid (AA). Treatment with either EA or HT alone visibly decreased the number of GFAP-positive cells, while combined treatment (AA + EA + HT) resulted in the most significant reduction in astrocyte reactivity.

Western blot analysis confirmed that GFAP protein levels were significantly elevated in the AA group (171.4 ± 59.53% of Control, *p* < 0.05), indicating enhanced astrocyte activation due to pain induction. EA treatment alone (AA + EA group) markedly reduced GFAP expression to 104.1 ± 33.75% of Control (*p* < 0.01 vs. AA group). HT alone (AA + HT group) partially decreased GFAP expression to 139.7 ± 23.83% of Control (*p* < 0.05 vs. AA group). The combined treatment (AA + EA + HT group) exhibited the most pronounced reduction in GFAP levels, decreasing them to 73.30 ± 21.63% of the Control (*p* < 0.0001 vs. AA group) and significantly different from EA or HT treatment alone (*p* < 0.05).

These results indicate that EA and HT effectively suppress spinal astrocyte activation, with a notable combined effect.

### 3.6. Combined EA and HT Treatment Significantly Improves Mechanical Hypersensitivity in the CFA-Induced Inflammatory Pain Model

**Figure 7:**
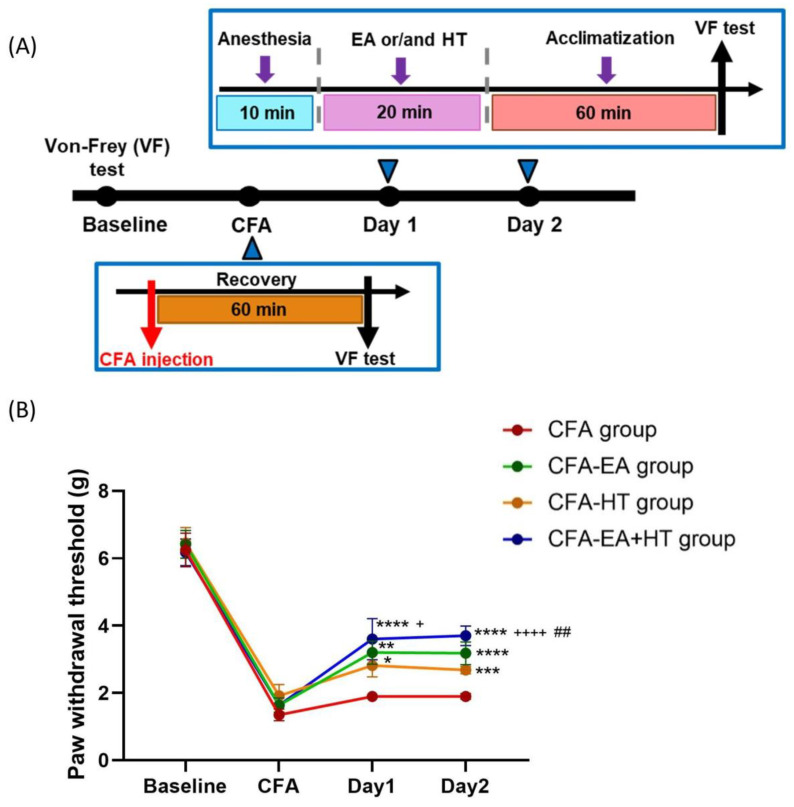
Evaluation of the analgesic effects of HT and EA, alone and in combination therapy, in a complete Freund’s adjuvant (CFA)-induced inflammatory pain model. (**A**) Timeline of CFA injection and von Frey test experiment. (**B**) Comparison of the effects of HT and EA, alone and in combination, on behavioral manifestations in the CFA model. Mechanical allodynia was assessed using the von Frey test. Data are presented as the mean ± SD (*n* = 4 in CFA group and *n* = 5 in other groups). Between-group comparisons were performed by one-way ANOVA, followed by the Holm–Šídák post hoc test at each time points (* *p* < 0.05, ** *p* < 0.01, *** p < 0.001, **** *p* < 0.0001 vs. CFA; ^##^ *p* < 0.01 vs. CFA + EA, ^+^ *p* < 0.05, ^++++^ *p* < 0.0001 vs. CFA + HT).

We assessed the analgesic effects of EA, HT, and their combination in CFA-induced inflammatory pain model, in which mechanical allodynia was assessed using the von Frey test [34,35] (Figure 7A). On the baseline day, all groups exhibited similar paw withdrawal thresholds (PWTs) (~6.32 g), indicating no pre-existing mechanical differences. Intraplantar injection of CFA caused a marked reduction in PWT (~1.64 g), confirming successful induction of mechanical allodynia. On days 1 and 2 post-CFA, PWTs in the CFA group remained unchanged. Among the treatment groups, EA (CFA + EA: 3.20 ± 0.34 g on day 1; 3.18 ± 0.33 g on day 2; *p* < 0.001 and *p* < 0.0001 vs. CFA, respectively) and HT (CFA + HT: 2.82 ± 0.34 g on day 1; 2.68 ± 0.11 g on day 2; *p* < 0.05 and *p* < 0.001 vs. CFA, respectively) each led to partial recovery of mechanical thresholds. Notably, the combined EA + HT treatment (CFA + EA + HT: 3.60 ± 0.61 g on day 1; 3.70 ± 0.29 g on day 2; both *p* < 0.0001 vs. CFA) produced the most pronounced analgesic effect. The CFA + EA + HT group also showed significantly higher PWTs than the CFA + HT group on both days (*p* < 0.05 on day 1; *p* < 0.0001 on day 2), and was significantly higher than the CFA + EA group on day 2 (*p* < 0.01), indicating a synergistic interaction between EA and HT in the CFA model (Figure 7B).

## 4. Discussion

**Figure 8:**
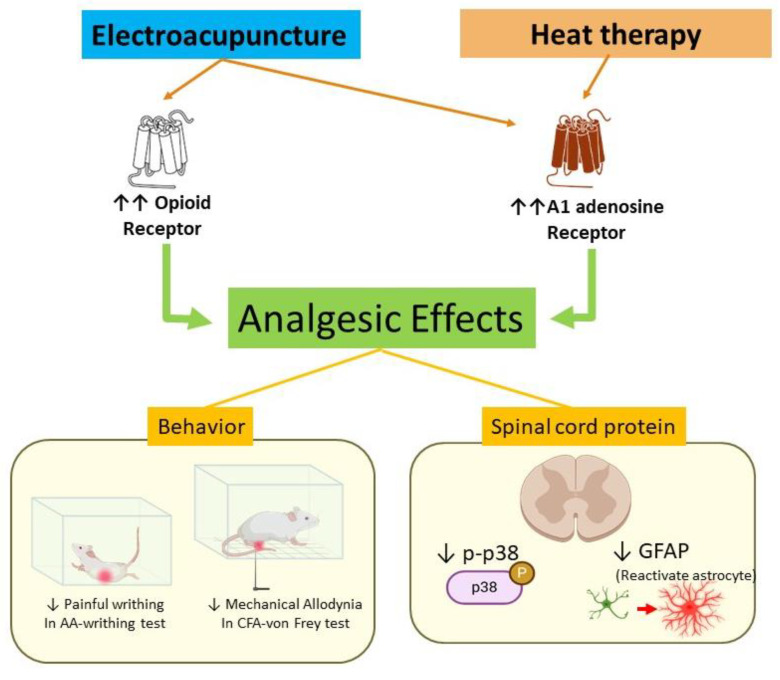
Proposed mechanism of electroacupuncture and heat therapy in a mouse model of inflammatory pain. Arrows in the diagram represent causal or modulatory relationships. Orange arrows indicate receptor activation induced by electroacupuncture or heat therapy. Green arrows represent downstream analgesic effects mediated through these receptors. Yellow lines illustrate behavioral and molecular outcomes observed following treatment. The red arrow highlights the role of GFAP as a marker of astrocyte activation, which is downregulated following intervention.

To the best of our knowledge, this is the first study to describe the analgesic effects of the combination of HT and EA in animal models of pain. While previous studies have explored the individual analgesic effects of EA, we provided experimental evidence of a possible synergistic effect when combined with HT. Our findings suggest that the combined treatment may be associated with mechanisms involving opioid receptors and the adenosine A1 receptor (Figure 8).

### 4.1. Analgesic Effects of HT

Several studies have demonstrated the analgesic effects of traditional moxibustion and its modern counterparts, such as heat therapy, in various conditions including osteoarthritis and visceral hyperalgesia [45,46]. Moxibustion’s analgesic properties are believed to be mediated by both temperature-related and non-temperature-related mechanisms [47]. In this study, we applied heat therapy at a controlled temperature of 45 °C and observed significant analgesic effects in the AA group (Figure 2). This is consistent with previous findings in neuropathic and chronic inflammatory pain models, where moxibustion at different temperatures (ranging from 37 °C to 52 °C) also produced pain relief [48].

It is important to note that, in traditional moxibustion therapy, the effectiveness of treatment can vary based on factors such as the type of moxa material and the distance from the skin [5]. In contrast, our use of an electric heating pad provided a constant, controlled heat source, which allowed us to exclude potential non-temperature-related influences like herbal effects, smoke, or infrared radiation [47]. Although excessive heat can activate nociceptors and trigger pain, therapeutic heat in the 40–45 °C range, as used in this study for 20 min, selectively activates thermosensitive channels without causing tissue damage. Importantly, due to heat conduction and tissue buffering, the temperature delivered to the skin remains sub-noxious despite the surface temperature of the heat source [49,50]. As a result, the analgesic effects observed in this study can be attributed solely to the controlled application of heat, which we hypothesize contributed to the reliable and reproducible therapeutic effect of HT.

### 4.2. A Synergistic Analgesic Effect of EA and HT

This study further investigated the combined effects of EA and HT in treating inflammatory pain induced by AA injection. Both EA and HT, when administered separately, significantly reduced the number of writhes in the writhing test, demonstrating their individual analgesic effects (Figure 1). Notably, the combination of EA and HT produced an even greater reduction in writhing, suggesting a synergistic analgesic effect. To further validate these findings, we employed the complete Freund’s adjuvant (CFA)-induced inflammatory pain model, in which CFA—a suspension of inactivated mycobacteria in mineral oil—is injected into the hind paw to induce localized inflammation and mechanical hypersensitivity. Mechanical allodynia was assessed using the von Frey test, which measures paw withdrawal thresholds in response to calibrated mechanical stimuli [34,35]. In this model, both EA and HT significantly alleviated mechanical allodynia, with their combination yielding the most pronounced analgesic effect (Figure 7).

Astrocyte is a type of glial cell found in the central nervous system (CNS). Astrocytes play an important role in maintaining homeostasis, protecting neurons, and regulating inflammatory responses in the brain. In response to injury, infection, or inflammatory conditions, astrocytes in the central nervous system (CNS) can become activated, contributing to pain sensitization through several mechanisms [51,52]. In support of our behavioral data, we observed molecular changes in astrocyte activation, which is known to be mediated by p38 MAPK signaling. Both EA and HT alone significantly reduced the upregulation of GFAP (a marker of astrocyte activation) and phosphorylated p38 (p-p38) levels. These findings align with previous studies showing that EA can modulate inflammatory responses through astrocyte-related pathways [40,53].

Moreover, the combination of EA and HT resulted in a striking downregulation of both GFAP and p-p38, more so than either treatment alone. This suggests that the two therapies, when combined, might exert a synergistic effect at the molecular level, inhibiting astrocyte activation and inflammation more effectively than either treatment individually. Although the exact pathways involved in this interaction remain unclear, our results strongly imply that the analgesic effects of the combined therapy involve the opioid receptor and adenosine A1 receptor.

### 4.3. Role of Opioid Receptors in EA and HT Analgesia

Opioids are powerful pain relievers that act on the nervous system to reduce pain perception [54]. A key finding in our study is that naloxone, an opioid receptor antagonist, partially reduced the analgesic effect of EA, indicating that opioid receptors are involved in EA’s mechanism, but not exclusively. This partial reduction suggests that EA may also activate other, non-opioid pathways, such as adenosine receptor-mediated analgesia, which has been previously reported in acupuncture studies [55]. In contrast, naloxone did not reverse the analgesic effect of HT, suggesting this treatment is independent of the opioid pathway and different from EA’s mechanism.

### 4.4. Modulation by A1 Adenosine Receptor: A New Insight of Heat Therapy-Induced Analgesic Effect

Another novel finding is that the combinations of HT/EA with caffeine (a non-selective adenosine receptor antagonist) or DPCPX (an adenosine A1 receptor antagonist) resulted in diminished analgesic effects compared to these treatments alone. Caffeine is often used to enhance analgesic responses in other contexts, yet it appears to counteract HT/EA’s pain-relieving effects in this model. This suggests that the adenosinergic system plays a crucial role in HT/EA-mediated analgesia. The findings were further confirmed by DPCPX pretreatment. The role of adenosine A1 receptor in EA analgesia was first reported in a CFA animal model by Goldman et al. in 2011 [31]. Adenosine A1 receptor antagonists or adenosine A1 knockout diminish the acupuncture analgesia in mice [31]. Moxibustion at ST36 triggers local ATP release; blocking ATP hydrolysis with ARL67156 enhances its analgesic effect, whereas accelerating ATP breakdown with ATPase reduces it, demonstrating that replenished interstitial ATP is a key mediator of moxibustion-induced pain relief [32]. However, the direct communication between moxibustion, as well as other heat therapy applications, and A1R, has not yet been reported.

The A1 adenosine receptor plays a critical role in pain inhibition, particularly in controlling both acute and chronic pain. A1AR activation causes hyperpolarization (increased negative charge inside neurons), making it more difficult for pain signals to be transmitted. This reduces the excitability of neurons involved in pain pathways. This activation can reduce the release of these mediators, including TNF-α, IL-1β, and IL-6, which helps in managing pain caused by inflammation [56].

The differential interactions of HT with naloxone, caffeine, and DPCPX highlight the complexity of pain pathways and the need for careful consideration when combining HT with pharmacological agents in clinical settings. These findings suggest that, while HT may be a promising non-invasive analgesic intervention, its effectiveness can be influenced by the concurrent use of specific drugs that modulate adenosine signaling. This is particularly relevant in pain management, where polypharmacy is common, and HT is often used as a complementary treatment.

Moreover, our study proved that EA or HT, and EA + HT decreased the upregulation of GFAP, manifesting the inhibition of astrocytes. A1AR is present not only in neurons but also in astrocytes. When activated, A1AR on astrocytes can reduce the release of pro-inflammatory cytokines and reactive oxygen species (ROS), thus helping to control neuroinflammation. A1AR activation inhibits astrocyte activation (or reactivity), reducing the inflammatory response in the CNS [57]. This is critical in conditions such as neuropathic pain, stroke, and Alzheimer’s disease, where astrocyte reactivity contributes to neuronal damage [58].

### 4.5. Limitations

This study employed the acetic acid-induced writhing test, or CFA-induced hind paw mechanical allodynia test, which are well-validated models for acute inflammatory pain. While this model is widely used for early phase analgesic screening due to its sensitivity and reproducibility, it does not fully represent the complexity of chronic inflammatory or neuropathic pain, which often involves central sensitization, glial activation, and prolonged neuroimmune interactions [34,35]. Additionally, this study focused on immediate analgesic effects, with molecular and behavioral outcomes assessed only 2 h post-treatment. As such, the long-term efficacy, durability, and safety of combined EA and HT remain unaddressed. Furthermore, while we focused on adenosine A_1_ and opioid receptors, pain modulation involves broader pathways including TRPV1, Nav1.7, serotonin, dopamine, and pro-inflammatory cytokines. Microglial activation is known to play a key role in pain, and our previous study in a dental pulp injury model demonstrated ionized calcium-binding adaptor molecule 1 (Iba1) upregulation [53]. As noted in the work by Donnelly et al. (2020), microglial activation often precedes astrocyte activation during the transition from acute to chronic [51]. However, while we assessed astrocyte activation, we did not evaluate microglial markers such as Iba1, limiting our ability to fully characterize glial involvement. Future studies using chronic pain models (e.g., spared nerve injury, chronic constriction injury) should also include both astrocytic and microglial markers, and incorporate broader pharmacological or genetic approaches, to further elucidate the therapeutic mechanisms effects of EA and HT and assess their sustained clinical potential.

### 4.6. Clinical Implications

We selected ST36 (Zusanli) based on its established role in abdominal pain relief and well-characterized anti-inflammatory mechanisms [59]. While ST36 served as a standardized acupoint in this study, clinical practice allows for flexible acupoint selection tailored to individual needs, and future studies may explore additional or combined points, such as SP6 or CV12 for visceral pain [60,61] and LI4 for orofacial pain [40].

The combination of EA and HT, inspired by traditional acupuncture and moxibustion, offers enhanced standardization and mechanistic clarity. Compared to moxibustion, which is time-consuming and produces smoke [62,63], our method offers a clean, controlled, and reproducible thermal stimulus. In clinical settings, this approach can be translated into practical applications by combining electroacupuncture (EA) with warm compresses or infrared light, either prior to or during EA treatment. EA + HT offers potential advantages over pharmacological treatments, including fewer systemic side effects, low dependency risk, and suitability for patients intolerant to medications. EA is minimally invasive, and HT is non-invasive, making the combination generally well tolerated. However, challenges include protocol standardization, practitioner training, patient variability, and the need for repeated sessions. Despite these, EA + HT represents a promising integrative pain management strategy warranting further clinical research.

### 4.7. Conclusions

In summary, both heat therapy (HT) and electroacupuncture (EA) produced significant analgesia in our acetic acid-induced writhing model, and their combination yielded even greater pain relief. At the molecular level, HT and EA each downregulated GFAP expression and inhibited p38 MAPK phosphorylation—an effect that was most pronounced when the two therapies were combined—indicating synergistic suppression of astrocyte activation and inflammation. These analgesic effects were further confirmed in a complete Freund’s adjuvant (CFA)-induced inflammatory pain model, where EA and HT individually alleviated mechanical allodynia, and their combination produced superior improvement in withdrawal thresholds. This study contributes to the growing body of evidence-based integrative pain research by identifying the distinct roles of adenosine A_1_ receptors in HT and EA, and the partial involvement of opioid receptors in EA. This mechanistic insight moves the field from observational outcomes to a receptor-targeted framework for integrative analgesia.

## Data Availability

The datasets used and/or analyzed during the current study are available from the corresponding author upon reasonable request.

## Abbreviations

AA	Acetic acid
CNS	Central nervous system
CFA	Complete Freund’s adjuvant
EA	Electroacupuncture
HT	Heat therapy
p-p38	Phosphorylated p38
MAPK	Mitogen-activated protein kinase
GFAP	Glial fibrillary acidic protein
DPCPX	1,3-dipropyl-8-cyclopentyl xanthine
TCM	Traditional Chinese medicine
